# Green leaf volatiles, fire and nonanoic acid activate MAPkinases in the model grass species *Lolium temulentum*

**DOI:** 10.1186/1756-0500-7-807

**Published:** 2014-11-18

**Authors:** James E Dombrowski, Ruth C Martin

**Affiliations:** USDA-ARS, National Forage Seed Production Research Center, Oregon State University, 3450 SW Campus Way, Corvallis, Oregon 97331-7102 USA

**Keywords:** Fire, Green leaf volatile, Grass, Herbicide, *Lolium*, MAPK, Systemic

## Abstract

**Background:**

Previously it has been shown that mechanical wounding, salinity and heat activated a 46 kDa and 44 kDa mitogen-activated protein kinases (MAPKs) in forage related grasses. Forage and turf related grasses are utilized in diverse environments where they are routinely subjected to herbicides and exposed to fire and volatiles after cutting, however very little is known concerning the perception or molecular responses to these different stresses or compounds.

**Results:**

In the model grass species *Lolium temulentum (Lt)*, a 46 kDa mitogen-activated protein kinase (MAPK) was activated in the leaves within 5 min and a 44 kDa MAPK 15 min after exposure to green leaf volatiles released from grass clippings. When the tips of leaves of *Lt* plants were scorched by fire, the 46 kDa MAPK and 44 kDa MAPK were rapidly activated within 5 min and 20 min respectively in the treated leaf, and 15 min systemically in an adjacent untreated tiller after exposure to fire. Nonanoic acid (pelargonic acid), a component in herbicides used on grasses, activated a 46 kDa MAPK in the treated leaves within 5 min of exposure and 15 min in systemic tissues. At concentrations normally used in the herbicides, nonanoic acid was found to only weakly activate the 44 kDa MAPK after an hour in treated leaves, but strongly activated it in the systemic tillers 30 min after treatment. Acetic acid, HCl and NaOH also were found to activate these MAPKs in treated tillers.

**Conclusion:**

The rapid activation of these MAPKs to a wide range of stress stimuli, suggest that these MAPKs play a role in the perception and response to these stresses and compounds. The activation of the MAPK by green leaf volatiles indicates a role for these compounds in wound signaling in grasses.

**Electronic supplementary material:**

The online version of this article (doi:10.1186/1756-0500-7-807) contains supplementary material, which is available to authorized users.

## Background

The most common abiotic stresses forage and turf related grasses are exposed to in the field are drought, herbicides, fire and grazing/cutting. Drought stress is and has been the most extensively studied of these stresses [1–8]. However much less is known on the molecular responses to the various components in herbicides, to fire and from grazing or cutting in forages and turf related grasses.

Herbicides are pervasively used around the world and the most extensively used herbicide is glyphosate [9, 10]. Glyphosate is a broad-spectrum herbicide that disrupts aromatic amino acid production by inhibition of the enzyme 5-enolpryruvylshikimate-3-phosphate synthase. There has been a great deal of research on the biochemical and molecular responses to glyphosate application, such as the genome wide profiling and analysis of miRNAs and transcriptomes in the forage grass tall fescue [11]. The molecular and physiological mechanisms of glyphosate resistance in grasses, such as *Lolium,* is also being vigorously investigated [12–16]. However much less is known on the other components utilized with glyphosate in its different formulations. Pelargonic acid is a nine-carbon fatty acid that is commonly used in conjunction with glyphosate [17]. It is a contact non-selective herbicide that disrupts intercellular pH and membrane integrity resulting in rapid cell death [17, 18]. While some physiological analyses have been done, very little is known on the plant’s molecular response to this compound. In rice, a cytochrome P450 gene was isolated from rice and expressed in yeast, and the enzyme was shown to catalyze the NADPH-dependent (**ω-**1)-hydroxylation of pelargonic acid, reducing its herbicidal activity, suggesting that it may participate in detoxification of the contact herbicide [19].

Fire is another type of stress grasses are exposed to, grass fields are routinely subjected to prescribed burning as a management practice and wildfires occur regularly in rangelands [20–25]. There has been a great deal of research investigating the ecological impact of fire on soil quality and composition, regrowth of grasses and plants, the effects of fire on composition of plant communities and species succession, disease and pests as well as the economic factors associated with burning of grass fields and rangelands. However, despite grasses frequent exposure to fire, there is virtually nothing known on the molecular responses to fire related stress in grasses or most other plant species. In tomato leaves, exposure to flame damage resulted in the activation of proteinase inhibitors genes locally and systemically [26, 27]. Furthermore the research suggested that in addition to chemical signals the systemic signalling had hydraulic and electrical components. Recently, it was reported that prescribed burning in pine was shown to affect the composition of secondary metabolites in the needles [28].

Forage and turf grasses are utilized and exposed to a wide range of environmental conditions in diverse geographical regions of the world. In addition to being exposed to various environmental stresses, these grasses are also routinely subjected to mechanical wounding and grazed upon by animals. Currently there is very little information available on the wound response in forage and turf grass species. Recently a few wound related signalling components have been identified in forage and turf grasses; a wound induced oxidative burst was shown in ryegrass lead blades [29], and a MAPK was rapidly activated within 5 minutes of wounding, both locally and systemically [30], and temperature was also shown to affect the wound activation of the MAPKs [31]. In tufted hairgrass, mechanical wounding was shown to release a characteristic complement of green leaf volatiles, however this blend of volatiles was significantly different than the profiles released when plants were treated with jasmonic acid [32]. In plants, grazing herbivores, pathogens, feeding insects, and mechanical wounding result in the release of a variety of volatiles for inter- and intra- plant communication as well as for signalling to other organisms, such as insects [33–35]. Different complements of volatile compounds are released from different stimuli indicating specific functions or roles, however the exact function for many of theses compounds is still not well understood.

Plants are exposed to a wide array of stresses and utilize multiple signaling pathways and signals to mediate their response to these stresses [36–40]. Many of these signaling pathways utilize common components to transmit the signals. An important conduit for stress signals are mitogen-activated protein kinase (MAPK) modules, which consist of three functionally linked kinases. MAPKs are activated sequentially through phosphorylation by MAPK kinases (MAPKKs or MEKs), which in turn are activated by MAPKK kinases (MAPKKK or MEKKs or MP3Ks). In response to a particular stress or combination of stresses, MAPKs and other signal transduction pathways will activate specific downstream targets, such as transcription factors, to elicit a specific response [41–44].

The various components of these MAPK cascades have been shown to enhance or improve tolerance to abiotic and biotic stresses [43–45]. While MAPKs in monocot plants are not as well characterized as the *Arabidopsis*, tobacco, and tomato MAPKs [45], a better picture is now emerging for rice [46, 47], maize [48–51] and wheat [52] MAPKs. Recently a survey of the *Brachypodium* genome, a model cereal related grass species, revealed 16 MAPKs genes. The analysis of these genes showed varying degrees of expression in different tissues, and transcriptional activation to various abiotic stresses [53]. In forage and turf related grasses, it was shown that mechanical wounding rapidly activated a 46 kDa and a 44 kDa MAPK in five different forage related grass species as well as *Brachypodium*[30]. In the model grass *L. temulentum*, the *Lt* p46 MAPK was activated by wounding, both locally and systemically [30], and the *Lt* p46 and p44 MAPKs were activated by salt and heat stress [31].

Currently very little is known concerning the perception or molecular responses to stresses or compounds that forage and turf grasses are routinely subjected to, such as herbicides, volatiles after cutting, and exposure to fire. We report here that volatiles released from cut grass rapidly activated a 46 kDa and a 44 kDa MAPK in the model forage grass species *L. temulentum*. Furthermore these MAPKs were also activated both locally and systemically in untreated tillers in plants with leaves exposed to fire or to nonanoic acid, an active component found in herbicides.

## Methods

### Plant materials

*Lolium temulentum* L. (Darnel ryegrass) cv. Ceres seeds were planted in 2 × 1.5 × 2 inch pots (1 plant/pot) containing SB40 Sunshine Growing Mix (Sun Gro Horticulture, Canada). Plants were fertilized weekly using Technigro 20-18-20 all-purpose fertilizer (Sun Gro Horticulture, Canada). Plants were grown under 12 h photo-periods at 21°C d and 17°C night in Conviron E15 and PGV 36 (Conviron, Winnipeg, Canada) growth chambers.

### Stress treatments

Plants were grown in flats for three to four weeks in a growth chamber, and then individual pots were separated and moved to a different growth chamber and allowed to rest overnight prior to treatment. The treatment duration for different compounds or stresses and the intervals of sample collection are described in their corresponding figures. Control plants were untreated and watered normally. All plant tissues were collected at the times indicated in the figure after treatment, placed in foil packets, immediately frozen in liquid nitrogen and stored at -80°C until processed.

#### Herbicide

The solutions containing the compounds (see below) as described in the figures, were applied directly onto the tiller using a 0.75 inch soft white bristle stencil brush or by spraying a mist onto the plant using a hand held spray/mister. Both application methods gave similar results for the various treatments. Compounds/herbicides used: Scythe Herbicide (Mycogen Corp CA USA) active ingredients: pelargonic acid 57%, related fatty acids (C6-C12) 3.0%, was diluted 1 to 25 mL of 0.1% or 0.2% Tween 20; Weed B Gon Max (Ortho USA) active ingredients: anime salts of the following 2-methyl-chlorophenoxyacetic acid 13.72%, 3,5,6,-trichloro-2-pyridinyloxyacetic acid 1.56% and Dicamba 1.35%, was diluted 1 to 64 mL with 0.1% or 0.2% Tween 20. Roundup Promax (Monsanto USA) active ingredients: glyphosate 48.7% was diluted 1 to 24 mL with 0.1% or 0.2% Tween 20; Roundup weed and grass killer - Ready to use plus (RdUp +) (Monsanto USA) active ingredients: glyphosate 2.0%, pelargonic acid and related fatty acids 2.0% was used as is without diluting. Glacial Acetic Acid (cat# 9508–03; JT Baker, USA), Hydrochloric Acid (cat# A144^C^-212; Fisher Scientific, USA), Linoleic Acid (cat# L1376; Sigma, USA), Linolenic acid (Cat# L2378; Sigma, USA), Sodium Hydroxide (cat# 7708; Malliinckrodt, USA) and Tween 20 (cat# P-7949; Sigma, USA). All solutions except Roundup: Weed and Grass Killer were diluted into 0.1% or 0.2% Tween 20, which acts as a surfactant that assists solutions adhesion to the leaf tissue, at the concentrations or dilutions described.

#### Fire

An 8–10 cm span of the tip of the main leaf of the treated tiller was scorched using a directed flame from a propane torch for a duration 1–2 seconds. In addition to the main tiller leaf, the tips (2–4 cm long) of auxiliary leaves in the vicinity were also scorched by the flame.

#### Green leaf volatiles

Freshly mowed grass clippings (*Lolium perenne*) were placed into the bottom (approximately 1 inch deep) of three 10 inch tall × 9 inch diameter Nalgene clear polycarbonate cylinders fitted with non air tight lids. The clippings were placed into the cylinders 3–5 minutes after mowing and supplemented with clippings generated from two 4-week-old *Lt* plants. The cylinders (with lids) containing the grass clippings were placed into a Conviron E15 growth chamber under light. Into each cylinder was placed 3 pots, each containing a three week-old *Lt* plant. The time delay between placing the grass clippings into the cylinder and the addition of the test plants was 8–10 minutes. Plants were exposed to volatiles emitted from the grass clippings by incubating the plants in the closed (non-air tight) cylinder in the growth chamber under light at the times indicated. The treated plants were removed from the cylinders at the times indicated by taking off the lid, removing the pot containing the treated plant from the cylinder and placing the lid back onto the cylinder, then immediately collecting and freezing the tissue: Cylinder #1 time points 3 and 15 minutes; Cylinder #2 time points 5, 20 and 45 minutes Cylinder #3 time points 10, 30 and 60 minutes. The time course for the control plants was performed as described above except there was no grass clipping contained in the cylinders.

### Sample preparation and immunoblot analysis

Sample preparation was performed as described [30]. Protein extracts (50 μg of extract/time point) were fractionated on 10% SDS-PAGE gels (1 mm thickness). The separated proteins were electro-transferred onto Immobilon-P (Millipore) membranes. Membranes were blocked for 1 hour at RT with 5% w/v BSA, 1× TRIS Buffered Saline (TBS), 0.1% Tween-20 with gentle shaking. The primary antibody [Phospho-p44/42 MAPK (Erk1/2) (Thr202/Tyr204) (D13.14.4E) XP™ Rabbit mAb (cat# 4370) from Cell Signaling Technology, Beverly MA] was diluted (1:2000) in 5% w/v BSA (fraction V), 1× TBS, 0.1% Tween-20. Membranes were gently shaken with the diluted antibody at 4°C overnight. The blots were then washed 3 times for 12 min/wash with 1× TBS, 0.1% Tween 20 at RT with gentle shaking, then the blots were incubated with the secondary antibody (Sigma Goat anti Rabbit Alkaline Phosphatase conjugate #A3937) diluted (1:2000) in 1% w/v BSA (Fraction V), 1× TBS, 0.05% Tween-20 at RT in the dark for 2 hours with gentle shaking. The blots were washed 3 times for 12 min/wash with 1× TBS, 0.1% Tween 20 at RT with gentle shaking, and then developed using a standard NBT/BCIP substrate mixture. In order to confirm the equal loading of protein per lane on the 10% SDS-PAGE gels and to verify the accuracy of the BCA protein assay, duplicate gels were run for a few randomly selected sample preps and were stained with Coomassie Blue. In addition, the consistency of protein transfer on to membranes was confirmed by periodically staining of the membrane with Ponceau S (see Additional file 1: Figure S1).

## Results

To determine if MAPKs are activated by compounds and conditions that forage and turf grasses are commonly exposed to in the field, three-to-four-week-old plants of the model grass species *L. temulentum*[54, 55] were exposed to herbicides, fire and green leaf volatiles. Phosphorylation of the tyrosine and serine/threonine residues in the activation motif TE/DY of many MAPKs is an indicator of their activation. To determine the phosphorylation/activation state of the MAPK, we used an anti-phospho-ERK (phospho-p44/42) MAPK antibody, which has been used in many studies to test MAPK activity in plants [30, 31, 56, 57].

**Figure 1:**
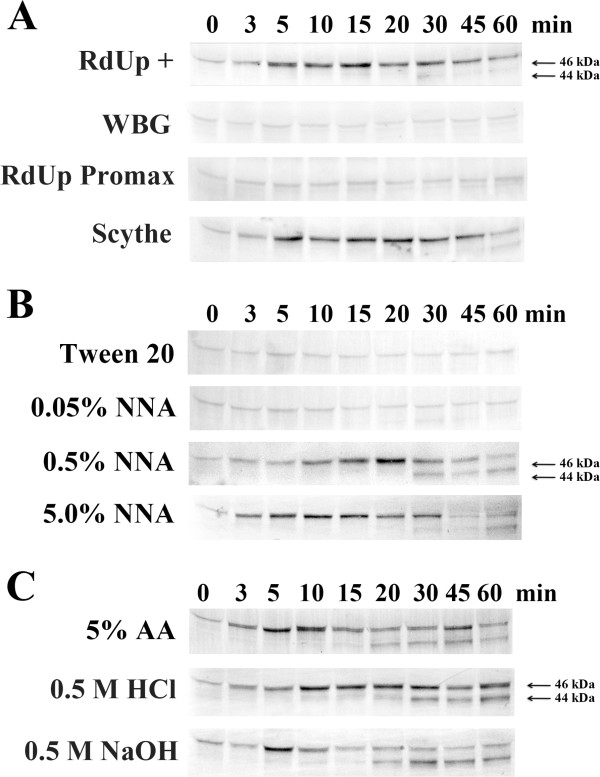
**Herbicide exposure results in the activation of a p46 and p44 MAPK in** ***L. temulentum*** **. (A)** The herbicides were painted directly onto the leaves of three-to-four-week-old plants using a soft bristle brush. RdUp+: "Roundup Weed and Grass Killer - Ready to use plus" was used directly from the bottle; WBG: "Weed-B-Gon Max concentrate" diluted 1 to 64 mL of 0.2% tween 20; RdUp Promax: "Roundup Promax concentrate" diluted 1 to 24 mL of 0.2% tween 20; Scythe: "Scythe Herbicide concentrate" diluted 1 to 25 mL of 0.2% tween 20. **(B)** Nonanoic acid (NNA) was sprayed onto the leaves of three-to-four-week-old plants at the concentrations indicated, all solutions contained 0.1% tween 20. **(C)** Acetic acid (AA), HCl and NaOH were sprayed onto the leaves of three-to-four-week-old plants at the concentrations indicated, all the solutions contained 0.1% tween 20. One plant per time point was collected at the times indicated above for each treatment described. To determine MAPK activity, immunoblots of protein extracts were performed using anti-phospho-MAPK (Erk1/2) antibody. The experiments were independently repeated a minimum of three times. Representative blots are presented.

To determine if herbicides commonly used on grasses would activate MAPKs in *L. temulentum*, plants were treated with herbicides at manufacturer’s recommended concentrations. The immunoblot analysis in Figure 1A shows the phosphorylation of MAPK in response to different herbicide formulations. No significant activation of a MAPK was observed in the aerial portion of plants treated with the herbicides Roundup Promax and Weed-B-Gon. However, the activation of a MAPK with an apparent molecular weight of 46 kDa (p46 MAPK) was observed in the aerial portion of plants within 5 min after exposure to the herbicides Roundup-Plus and Scythe. This activation began to wane after 45 min of exposure. In addition to the p46 MAPK, another MAPK with an apparent molecular weight of 44 kDa (p44 MAPK) was weakly activated within 30 to 60 min after herbicide treatment. In plants treated with Roundup Promax and Weed-B-Gon, there were no visible signs of damage after an hour of treatment, however the tissues exposed to Roundup-Plus and Scythe showed visible signs of damage within 15 minutes, with the leaves displaying some loss of turgor and after 30 to 45 min of exposure the treated leaves began to shrivel up. After 24 hours the treated tissues exposed to Roundup-Plus and Scythe had died back, while the plants treated with Weed-B-Gon continued to grow, and the plants treated with Roundup Promax appeared to grow slower and eventually yellowed and died 5 to 6 days post treatment.

The common components found in the Roundup-Plus and Scythe were pelargonic acid (nonanoic acid), a 9-carbon chain fatty acid, and other related short chained fatty acids. To further investigate if the predominant component of these herbicides, nonanoic acid (NNA) commonly known as pelargonic acid, was the compound activating p46 MAPK, we subjected *L. temulentum* plants to different concentrations of purified NNA. As shown in Figure 1B, when plants were sprayed with 0.1% tween 20 or 0.05% NNA in 0.1% tween 20, no activation of a MAPK was observed up to 60 min after the treatment. However, when plants were treated with a 10 fold increase in NNA (0.5%), the activated p46 MAPK was observed after 10 min of exposure, with maximum activation after 20 min and return to background levels after 60 min. A p44 MAPK was also observed to be weakly activated within the 30 to 60 min time frame. An additional 10 fold increase in NNA (5.0%) resulted in a rapid activation of the p46 MAPK 3 min after treatment, displaying a maximum activation after 10 min, which began to wane after 15 min and returned to background levels by 45 min. A p44 MAPK band was observed to be weakly activated after 60 min. The 5.0% NNA shows a similar p46 MAPK activation pattern to that observed with Roundup-Plus and Scythe in Figure 1A.

Previously it has been shown that when *Lt* plants were exposed to methyl jasmonate, MAPKs were not activated [30]. Since exposure to the short chain fatty acids did result in MAPK activation, we wanted to determine if linolenic acid, the precursor to jasmonic acid, a potent activator of many cellular responses, could activate the MAPKs. Linolenic acid (long chain poly-unsaturated fatty acid, 18:3) and linoleic acid (long chain unsaturated fatty acid, 18:1) were diluted to 10 mM concentrations in 0.1% tween 20, a concentration shown to activate wound response genes [58, 59] and sprayed onto 3-week-old *Lt* plants. Neither treatment resulted in the activation of the MAPKs (data not shown).Historically vinegar, approximately 5% acetic acid (AA), and other acids were used as herbicide treatments. Therefore we investigated if acetic acid or another acid, such as HCl, or a base such as NaOH, would activate the MAPKs when applied onto plants. As shown in Figure 1C, plants treated with 5% AA showed a rapid and strong activation of the p46 MAPK after 5 min, that waned after 10 min, and displayed a second transient activation after 45 min. The 44 kDa MAPK was weakly activated by AA after 20 min and remained constant to the end of the time course. HCL gave a strong prolonged activation of the p46 MAPK after 10 min, and a constant weak activation of the p44 MAPK after 30 min. NaOH had a transient activation of the p46 MAPK at 5 min and the 44 kDa MAPK showed a similar activation pattern as the other 2 treatments.

**Figure 2:**
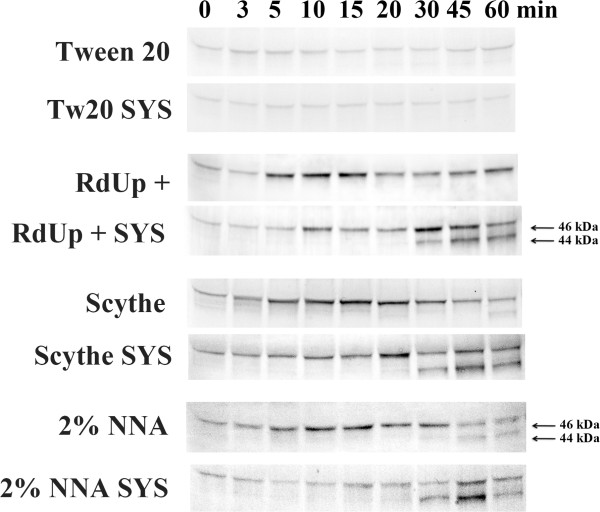
**Systemic activation of p46 and p44 MAPKs by nonanoic acid in** ***L. temulentum*** **.** The herbicides were painted directly onto the leaves of three-to-four-week-old plants using a soft bristle brush. The control plants were treated with a solution of 0.1% tween 20. RdUp+: "Roundup Weed and Grass Killer - Ready to use plus" was used directly from the bottle (2% NNA and related fatty acids); Scythe: "Scythe Herbicide concentrate" diluted 1 to 25 mL in 0.1% tween 20 (final concentration of 2% NNA and related fatty acids). Nonanoic acid (NNA) was diluted to 2% by diluting it 1 to 50 mL in 0.1% tween 20. One plant per time point was collected at the times indicated above for each treatment described, the tissues from the treated tiller and systemic tiller (SYS) were collected and processed separately. To determine MAPK activity, immunoblots of protein extracts were performed using anti-phospho-MAPK (Erk1/2) antibody. The experiments were independently repeated a minimum of three times. Representative blots are presented.

Recently it was shown that mechanical wounding rapidly activated a 46 kDa and a 44 kDa MAPK in six different grass species; and in *L. temulentum*, the *Lt* p46 MAPK was rapidly activated within 5 minutes of wounding, both locally and systemically [30]. We wanted to investigate if the herbicides containing NNA could also activate the MAPKs systemically in an adjacent untreated tiller. As shown in Figure 2, control plants displayed no significant activation of the MAPKs in treated or systemic tissues when treated with 0.2% tween 20. In plants exposed to 2.0% NNA or the herbicides containing it (a relative concentration of 2.0%), all three solutions containing NNA displayed similar patterns of activation of the p46 MAPK in the treated leaves and displayed systemic activation of both MAPKs in the untreated systemic tiller. The p46 MAPK was strongly activated by all three treatments in exposed tissues, however in systemic tissues its activation was less pronounce and delayed. Furthermore the systemic activation of p46 MAPK was observed to be more intense in the two herbicide treatments as compared to its weak activation in the NNA treatment. Surprisingly the p44 MAPK was not significantly activated in the treated tillers in both herbicide treatments and only weakly activated at 45 and 60 min in the NNA treatment. However, in all three treatments, the p44 MAPK was found to be activated systemically 30 min after treatment, displaying a maximum activation after 45 min with a reduced activity observed at 60 min. While the overall pattern of activation was similar between the three different treatments, there was variation in the intensity of activation of the MAPKs at the various time points. It should be noted, that while the relative concentration of NNA (2.0%) was similar between the three treatments, the two herbicide formulations also contained related short chain fatty acids as well as other "inert" ingredients that were not present in the NNA treatment. The presence of these additional components may have contributed to the variations in MAPKs activation patterns observed between the three treatments.

**Figure 3:**
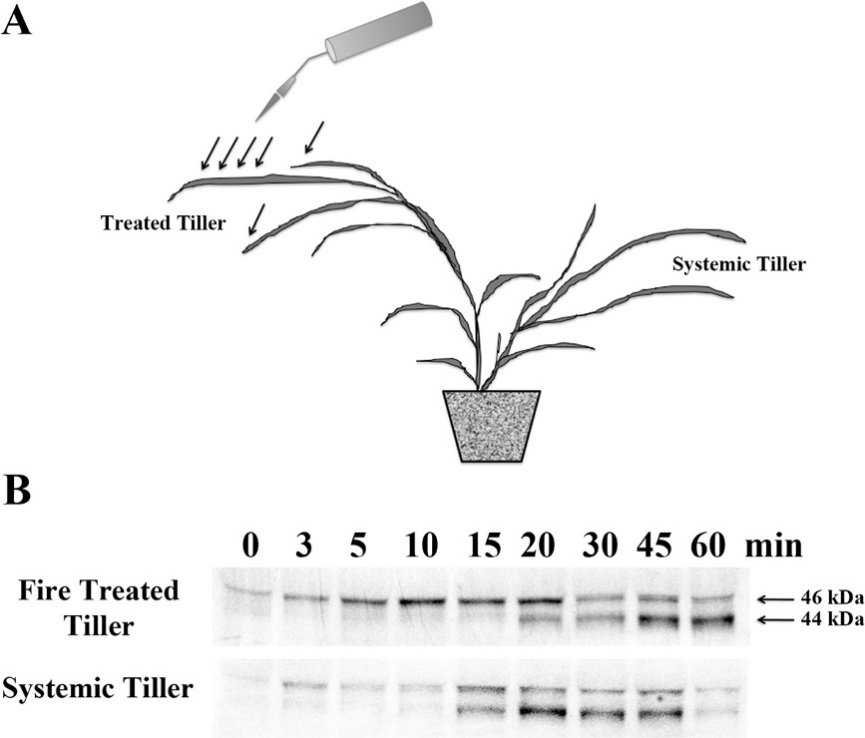
**Fire stress activation of the p46 and p44 MAPKs locally and systemically in** ***L. temulentum*** **.** Three-week-old plants were exposed to direct flame from a propane torch for a duration 1–2 seconds. **(A)** The region of the leaves and tiller exposed to the flame are indicated by arrows in the illustration. **(B)** One plant per time point was collected at the times indicated, the tissues from the fire treated tiller and untreated systemic tiller were collected and processed separately. To determine MAPK activity, immunoblots of protein extracts were performed using anti-phospho-MAPK (Erk1/2) antibody. The experiments were independently repeated a minimum of three times. Representative blots are presented.

To determine if the p46 and p44 MAPKs are activated by exposure to fire, four-week-old *Lt* plants at the two-to-three-tiller stage were exposed to direct flame for 1–2 sec, and the tips of the leaves on largest tiller were scorched as illustrated in Figure 3A. As shown in Figure 3B, there was a strong and rapid activation of the p46 MAPK in the stressed tiller after 5 min of exposure to flame that increased in intensity, began to wane after 20 min, and returned to background levels by 30 min. The p44 MAPK was also found to be activated in the flame exposed tillers after 20 min, and was found to increase in intensity at 45 min and 60 min post-exposure. In the systemic tiller(s), the p46 and p44 MAPKs were both shown to be activated after 15 min and returned to background levels after 60 min, however the p46 MAPK’s activation was observed to be less pronounced than the p44 MAPK.

**Figure 4:**
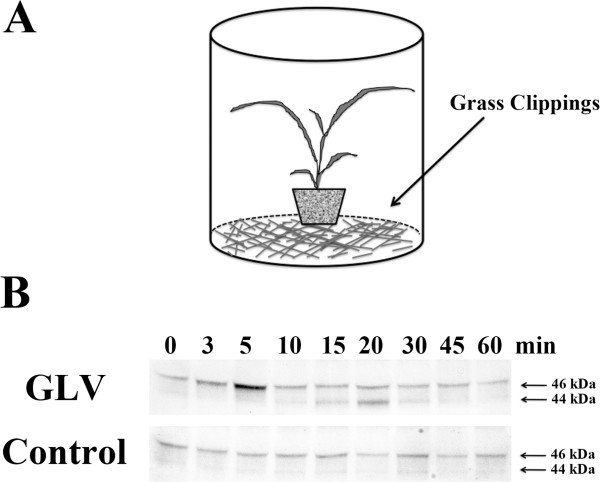
**Exposure to green leaf volatiles activated p46 and p44 MAPKs in** ***L. temulentum.*** **(A)** Three-week-old plants at the one tiller stage were placed into a closed clear (non-air tight) cylinder with freshly mowed grass clippings (see illustration). Control plants were placed into the cylinders without any grass clippings present. **(B)** One plant per time point was collected at the times indicated. To determine MAPK activity, immunoblots of protein extracts were performed using anti-phospho-MAPK (Erk1/2) antibody. The experiments were independently repeated three times. Representative blots are presented.

In tufted hairgrass, tissue damage has been shown to be accompanied by the release of volatile compounds [32]. Most grasses are routinely exposed to damage by grazing animals or cutting. Therefore in order to determine if volatiles released from cut grass could activate MAPKs, 3-week-old *Lt* plants were placed in a closed clear (non-air tight) cylinder with freshly mowed grass clippings (Figure 4A). Figure 4B shows that when *Lt* plants were incubated with freshly cut grass clippings, the p46 MAPK displayed a strong, rapid, but transient activation after 5 min that quickly returned to background levels after 10 min exposure. The p44 MAPK was weakly activated after 15 min, increasing after 20 min, and returning to background levels after 30 min of exposure.

## Discussion

Unlike most plants including cereal grasses, forage and turf grasses are routinely being cut or grazed upon by animals. Currently there is very little information available on the wound response in forage and turf grass species. Previously, we have shown that mechanical wounding in six different grass species rapidly activated a p46 and p44 MAPK. In the model grass *L. temulentum*, the p46 MAPK was also shown to be systemically activated [30]. While mechanical wounding, exposure to fire and NNA caused tissue damage to the tiller or leaf, the type of damage inflicted from each was significantly different. In the tillers exposed to fire damage and mechanical wounding, the stimuli that caused the damage was transient in nature, the tiller recovered and was still able to grow. However in the case of the herbicide treatment, the damage inflicted was more severe due to prolonged exposure that resulted in the die off of the tiller. Mechanical, fire and chemical induced tissue damage all appear to activate the same p46 and p44 MAPKs, however only fire and NNA displayed systemic activation of the p44 MAPK, which was not observed systemically after mechanical wounding [30]. The signal generated from the damage presumably activates two MAP kinases with different activation kinetics. In all cases, the p46 MAPK is rapidly and strongly induced within 3–5 min after treatment, while the activation of the p44 MAPK is always delayed for 15–30 after treatment. The difference in the kinetics of the MAPKs may be due to different modes of activation (transcriptionally or post-transcriptionally regulated), different signaling pathways leading to their activation, or different threshold levels for the activating signal.The p44 MAPK was strongly activated both locally and systemically after fire stress. However in NNA treated plants, the p44 MAPK was only weakly activated locally but strongly activated systemically. Plant tissues directly exposed to herbicides containing NNA (Figure 2) experienced rapid cellular die off, which may have prevented or reduced the activation of the p44 MAPK, however in systemic tissues where there was no actual tissue damage the p44 MAPK was able to be activated. Furthermore the inert components present in the herbicide formulations may also enhance the damage caused by NNA resulting in more rapid cellular death (Figure 2). Evidence for this hypothesis can be seen in Figure 1B where at the highest concentration of NNA, the p44 MAPK activation was reduced, but at lower concentration of NNA the p44 MAPK is more strongly activated in the treated tissues. At this concentration, the treated tiller does recover slowly and grows with some visible damage evident.

The activation of MAPKs by NNA and not by glyphosate suggests that when studying the effects and the molecular response to a herbicide, each specific component in the herbicide formulation and the herbicide as a whole needs to be investigated in order to understand the overall effect of the application on the plant and how it will respond. Furthermore, it is not only the active ingredients that need to be investigated, since the so-called inert ingredients have the ability to alter molecular expression of genes within the plant. In *Arabidopsis*, the expression of 196 genes were significantly altered 1 hour after treatment with a surfactant used with herbicides [60]. The different components in the 3 herbicide formulations containing NNA may have contributed to the differences in the activation profiles of the p46 and p44 MAPK observed in Figure 2.

It appears that damage from mechanical, fire and herbicide stresses all activate the same MAPK cascades, however it is not known if the plant’s downstream response to the various forms of tissue damage is similar or different. Often, common signaling elements are utilized for perception and response, however other inputs and signals are utilized to tailor the proper response to a specific stress or stimuli. In plants, many signaling components have been shown to be activated or induced by small signaling molecules. In forage and turf grasses, a couple of signalling molecules induced by wounding have been identified, a wound induced oxidative burst was shown in ryegrass lead blades [29], and the release of green leaf volatiles (GLV) after mechanical wounding in tufted hairfgrass [32]. Previously, we showed that methyl jasmonate (MJ), a volatile signaling molecule that is a potent inducer of the wound response in dicotyledonous plants, was not able to activate, enhance or suppress the wound activation of the *Lt* p46 MAPK. However, the pretreatment of plants with MJ prior to wounding did accelerate and potentiate the wound activation of the p44 MAPK [30]. This result suggested that MJ specifically enhances p44 MAPK activity through an unknown mechanism. In contrast to MJ, GLV released from grass clippings did activate both the p46 and p44 MAPKs. The p46 MAPK was rapidly activated after only 5 min of exposure to the GLV, and the p44 MAPK after 15 min, which was similar kinetically to what was observed by direct mechanical wounding, fire or NNA treatments.

One might ask, how biologically relevant is the actual exposure levels of volatiles in the cylinder compared to what a grass plant may be exposed to in the field. If one was investigating insect damage, the release of volatiles from feeding would in all probability be miniscule compared to the levels found in the cylinder from the grass clippings. However, in the field when forage or turf grasses are cut, as in a lawn, or for collection of hay, or when harvesting seed and the plants are still green, the cut grass is normally left behind, and the levels of GLV released in localized areas could approach those found in the cylinder during our experiment. It should be noted that the concentration and duration of the GLV in the field would be impacted by environmental conditions such as wind, humidity, and temperature.

The GLV may have a dual role for inter- as well as intra-plant signaling. Even if plants are exposed to below threshold levels of GLV for activation of a stress response, this low level exposure may prime or sensitize the plant’s response system towards future stimuli and enhance the overall response to the stress [33, 61, 62]. Furthermore, it was recently demonstrated in *Arabidopsis* that plants could respond to long-term repeated exposures to subcritical amounts of GLV [63]. While mechanical wounding has been shown to release GLV in forage grass, volatiles release from damage due to herbicides and fire may also occur. It will be interesting to determine if the GLV blends released from these different types of plant injury are the same or different.

## Conclusions

We have shown that when grass plants are exposed to GLV, fire or NNA containing herbicides, it resulted in the activation of MAPKs locally and systemically. In treated tissues a MAPK was rapidly activated after 5 minutes, this is similar to the timeframe that was observed in plant tissues subjected to mechanical wounding [30]. The transient activation of the *Lt* MAPKs despite prolonged exposure to GLV from cut grass clippings suggests that a GLV induced signal is not the sole inducer of the MAPKs, since the duration of MAPK activation in plants from wound damage is much longer (20 to 30 minutes post wound) [30, 31] than is observed for GLV exposure alone. In addition, the amounts of GLV released due to the inflicted damage on a single plant would be quite low as compared to the amount released by the grass clippings. This data suggests that the damaged leaf tissue may release volatiles that potentially prime the nearby undamaged tissue and the adjacent undamaged tiller for the arrival of secondary signal(s), such as an oxidative burst [29, 64], electrical or hydraulic pulse [65, 66] or another signaling molecule [67–70], resulting in the activation of the MAPKs and other downstream elements.

Currently very little is known concerning the perception or molecular responses in forage related grasses to damage caused from exposure to herbicides, fire or volatiles after cutting/wounding, and how these responses affect the persistence and the long-term quality of the grass. To our knowledge, this is the first report detailing the activation of MAPKs by signals generated from exposure to green leaf volatiles, and the local and systemic activation of MAPKs resulting from plant damage caused by fire and nonanoic acid, a component commonly used in herbicides. The rapid activation of these MAPKs, suggest that they play a role in the perception and response to these particular stresses and compounds, and that the signal leading to their activation may have a volatile component. Furthermore, these MAPKs have been shown to be rapidly activated by a wide range of abiotic stresses including salt, wounding, heat, fire and chemicals [30, 31]; therefore they may have utility as markers to determine if a particular tissue has perceived the stress. Future research will be directed at identifying and comparing the types of volatiles released by damage caused from mechanical wounding, fire, and herbicides. In addition, it would be of interest to determine which of the volatiles released from the grass clippings are involved in the activation of the p46 and p44 MAPKs, what is being activated downstream of the MAPK cascade, and how the plant responds to this activation at the molecular or physiological level.

## Electronic supplementary material

Additional file 1: **Loading controls.** (TIFF 3 MB)

## Abbreviations

BCIP: 5-bromo-4-chloro-3′-indolyphosphate p-toluidine salt
BSA: Bovine serum albumin ERK, Extracellular signal regulated kinase
GLV: Green leaf volatiles
*Lt*: *Lolium temulentum*
MAPK: Mitogen-activated protein kinase
MJ: Methyl jasmonate
NBT: Nitro-blue tetrazolium chloride
NNA: Nonanoic acid
PAGE: Polyacrylamide gel electrophoresis
ROS: Reactive oxygen species
SDS: Sodium dodecyl sulfate
TRIS: 2-Amino-2-hydroxymethyl-propane-1,3-diol.
