# Supplementary figures and images for: Green leaf volatiles, fire and nonanoic acid activate MAPkinases in the model grass species Lolium temulentum
Source: BMC Res Notes. 2014 Nov 18;7:807. doi: 10.1186/1756-0500-7-807 (PMC4289168; doi:10.1186/1756-0500-7-807)

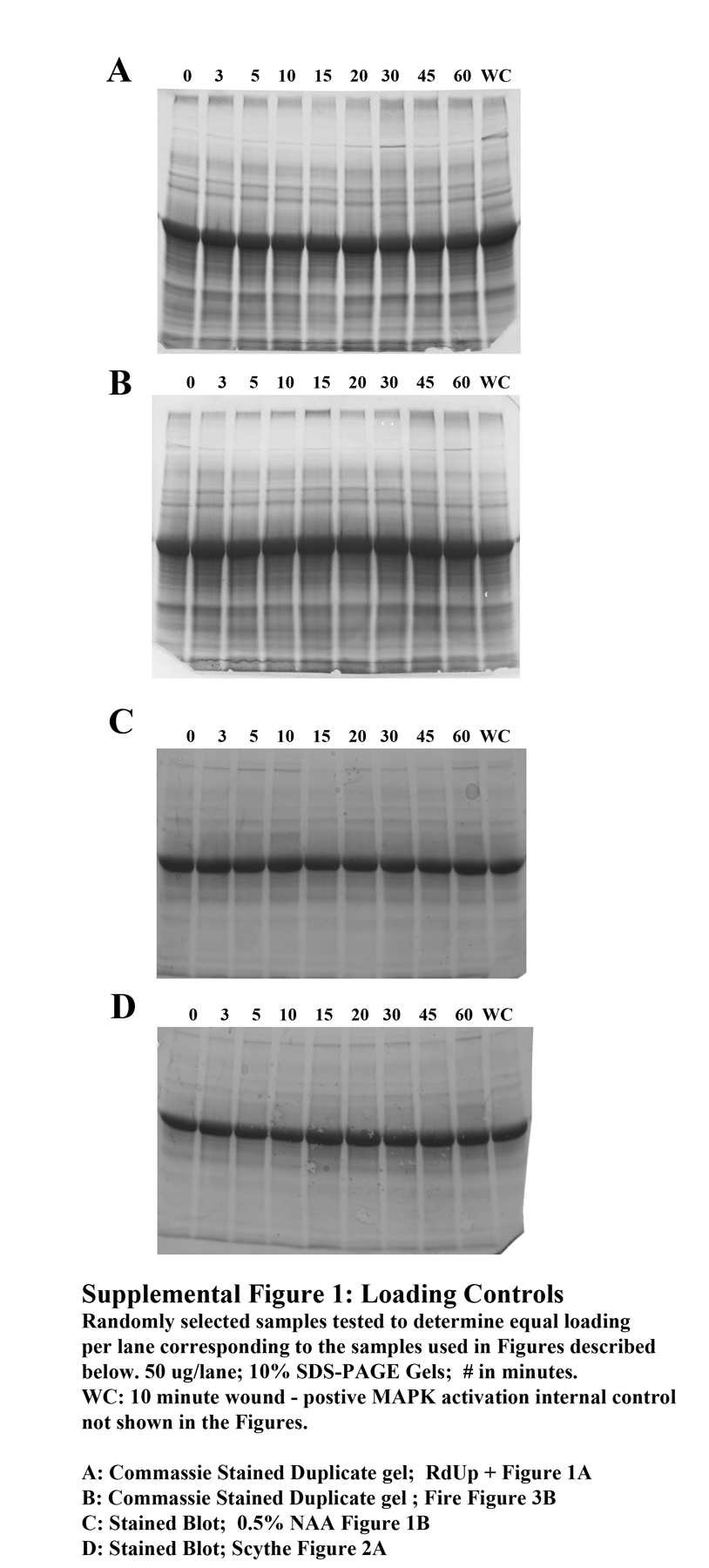

Supplement: Supplementary file 1 — Additional file 1: Loading controls. (TIFF 3 MB) [file 13104_2014_3439_MOESM1_ESM.tiff]
